# Sexual dimorphism in cognitive disorders in a murine model of neuropathic pain

**DOI:** 10.1186/s12993-019-0164-0

**Published:** 2020-01-04

**Authors:** Soonmi Won, Keebum Park, Hyoungsub Lim, Sung Joong Lee

**Affiliations:** 1 Department of Neuroscience and Physiology, Dental Research Institute, Seoul National University School of Dentistry, Seoul, Republic of Korea; 2 Interdisciplinary Program in Neuroscience, College of Natural Science, Seoul National University, Seoul, 08826 Republic of Korea

**Keywords:** Chronic neuropathic pain, Depression, Anxiety, Cognitive deficits, Sexual dimorphism

## Abstract

**Background:**

A sex-difference in susceptibility to chronic pain is well-known. Although recent studies have begun to reveal the sex-dependent mechanisms of nerve injury-induced pain sensitization, sex differences in the affective and cognitive brain dysfunctions associated with chronic pain have not been investigated. Therefore, we tested whether chronic pain leads to affective and cognitive disorders in a mouse neuropathic pain model and whether those disorders are sexually dimorphic.

**Methods:**

Chronic neuropathic pain was induced in male and female mice by L5 spinal nerve transection (SNT) injury. Pain sensitivity was measured with the von Frey test. Affective behaviors such as depression and anxiety were assessed by the forced swim, tail suspension, and open field tests. Cognitive brain function was assessed with the Morris water maze and the novel object location and novel object recognition tests.

**Results:**

Mechanical allodynia was induced and maintained for up to 8 weeks after SNT in both male and female mice. Depressive- and anxiety-like behaviors were observed 8 weeks post-SNT injury regardless of sex. Chronic pain-induced cognitive deficits measured with the Morris water maze and novel object location test were seen only in male mice, not in female mice.

**Conclusions:**

Chronic neuropathic pain is accompanied by anxiety- and depressive-like behaviors in a mouse model regardless of sex, and male mice are more vulnerable than female mice to chronic pain-associated cognitive deficits.

## Background

Neuropathic pain is chronic pathologic pain that develops due to lesions or dysfunctions in the nervous system. The prevalence of neuropathic pain in developed countries is estimated to be 2% to 3% [1] and is expected to increase during the next few decades as the populations of cancer survivors and obesity patients experiencing neuropathic pain increase. Chronic pain, including neuropathic pain, is often accompanied by affective disorders, particularly anxiety and depression, and cognitive impairment. Clinical studies have revealed that more than half of patients with chronic pain have major depression [2, 3]. The co-morbidity of depression and chronic pain has been also described in several studies using animal models, but a causal relationship has not been clearly demonstrated [4–7].

There are well-known sex differences in susceptibility to chronic pain. In general, females have a much higher prevalence of chronic pain [8]; however, the vast majority of animal research on pain has been performed using males [9, 10]. Of interest, recent studies using mouse models have shown a sex difference in the mechanisms of nerve injury-induced neuropathic pain. In male mice, activation of the spinal cord microglia is responsible for nerve injury-induced central sensitization [11]. However, in female mice, nerve injury-induced central sensitization is independent of microglial activation and is mediated by spinal cord-recruited T cells. In addition, intrathecal lipopolysaccharide administration induces spinal cord microglial activation and central pain sensitization only in male mice [12]. Therefore, spinal cord microglial activation is differentially involved in inducing chronic pain, which might underlie the sexual dimorphism in the susceptibility to chronic pain.

Of note, sexual dimorphism also manifests in clinical studies of affective and cognitive disorders such as major depression and anxiety. The prevalence of major depression in females is about twice that in males [13–15]. Also, more females have anxiety disorder than males [16–18]. Likewise, epidemiologic studies report that females make up almost two-thirds of patients with Alzheimer’s disease in the United States [19], even though males might have a greater risk for mild cognitive impairment [20, 21]. The comorbidity of depression and chronic pain is also higher in females than in males [8]. Considering the highly prevalent sexual dimorphism in affective and cognitive disorders, it is possible that susceptibility to chronic pain-associated affective and cognitive brain dysfunctions vary by sex, but that idea has not been formally addressed. In this study, we explored that possibility using a spinal nerve injury-induced neuropathic pain model, and we provide evidence of sexual dimorphism in chronic pain-associated cognitive disorders.

## Methods

### Animals

Male and female C57BL/6 J mice (8 weeks old) were used for this experiment. Food and water were provided ad libitum. Lights were maintained on a 12 h light–dark cycle. All experiments were performed in the light phase. All experiments were approved by the Institutional Animal Care and Use Committee of Seoul National University and conducted according to National Institutes of Health guidelines.

### Neuropathic pain model

Animal treatments were performed in accordance with guidelines from the International Association for the Study of Pain. A persistent pain model was induced using L5 spinal nerve transection (SNT), as previously described [22]. Briefly, under anesthesia with pentobarbital sodium (50 mg/kg, i.p.), an incision was made in the skin above the spinal processes at the L4-S2 level. The paraspinal muscles were separated, and the L6 vertebral transverse process was carefully removed with small scissors. The right L5 spinal nerve was exposed and carefully transected with small scissors. Then, 10% povidone-iodine solution was applied to the surgical site, which was closed in 2 layers with surgical staples. Sham-operated mice underwent removal of the L6 transverse process. All procedures were performed under sterile conditions. Animals that had been operated on were monitored on a warm pad during recovery.

### Behavioral tests

Mechanical allodynia was assessed by the von Frey test. Activity during the open field test, forced swim test, tail suspension test, Morris water maze, novel object recognition test, and novel object location test was video recorded and, except for the tail suspension test, automatically analyzed by a computer-operated tracking system. Investigators scored the tail suspension test by watching the videos.

#### von Frey test

Animals were habituated to the testing environment daily for 3 days. To measure mechanical allodynia, mice were placed in clear plastic chambers on an elevated table and allowed to acclimate for approximately 30 min. The up-down method was used with a set of von Frey filaments (0.02–2 g; North Coast Medical, Morgan Hill, CA, USA) to determine the 50% withdrawal threshold, as previously described [22].

#### Open field test

Mice were placed in a 40 × 40 cm field surrounded by a 30 cm high wall. Total distance traveled, time spent, and distance in the center and periphery were recorded. Data were collected over a 5 min period.

#### Tail suspension test

The tail suspension test was performed for a 5 min test session. Mice were suspended 60 cm above the floor in a visually isolated area using adhesive tape placed 1 cm from the tip of the tail, and their behavior was recorded over a 5 min test period.

#### Forced swim test

The forced swim test was performed with plastic cylinders (25 cm height × 10 cm diameter) filled with water (23 °C) to 5 cm from the top. Mice were placed in the cylinders, and their behavior was recorded over a 6 min test period. The recording period consisted of 2 min of habituation and 4 min of testing. Data acquisition and analysis were performed automatically using the SMART system (V3.0, Panlab, Barcelona, Spain).

#### Morris water maze test

The Morris water maze test was performed to assess spatial learning and memory, as previously described [23]. Mice were tested at the same time each day (1:00–4:00 p.m.) using a 100 cm diameter tank filled with 21 °C water. Four 60 s trials per day were performed for 5 days. After starting from a designated point, the mice had 60 s to find a 10 cm platform located 1 cm below the water surface made opaque with nontoxic paint powder. After reaching the platform or spending 60 s swimming, the mouse was left on the platform for 30 s and then returned to a cage for 10–15 min. Training was conducted by a visible trial test on the first day followed by a probe trial (60 s) on the sixth day using a video tracking SMART system that recorded latency, distance to reach the platform, and time spent in each platform quadrant.

#### Object location recognition test

Mice explored the same box for four consecutive days during the open field test. Training and testing were conducted as previously described [24]. The session consisted of habituation to an empty area and then three training sessions with two distinct objects. The arena and objects were wiped down after each session with 70% ethanol to remove olfactory stimuli. Following 5 min of training, mice were re-exposed to the arena for 5 min with one object displaced to a novel location. Testing sessions were video monitored, and object exploration times were scored off-line by an experimenter blinded to the object location recognition test. Interaction time and a discrimination index (new place-old place/total time) during the test session were scored.

#### Novel object recognition test

The novel object recognition test was performed using previously described methods [25] with minor modifications. On the first day of the experiment, each mouse was placed on one side of the open field box and allowed to freely explore for 5 min, which was repeated for 3 consecutive days. After familiarization, two identical objects were presented to each mouse for 5 min. After 5 min of exploration, one object was replaced with a novel object, and the mouse was allowed to explore for 5 min. The chamber and objects were cleaned with 70% ethanol between trials to remove olfactory stimuli. Results are expressed as the interaction time and discrimination ratio of time spent with the novel object to the total exploration time.

### Statistics

Statistical analyses were performed and graphs were made using GraphPad Prism 7.0 for Windows software (Software Inc., La Jolla, CA, USA). All data are expressed and plotted as the mean ± SEM. *P* < 0.05 and *P* < 0.01 were considered statistically significant. Statistical tests between groups were made using unpaired t-testing for all behavioral tests except the Morris water maze. Morris water maze values were analyzed using two-way ANOVA with repeated measures. For the power calculation in novel object location and novel object tests, we performed a priori analysis using G* Power 3.1 software with α = 0.05 and power (1 − β) = 0.80.

## Results

### SNT-injured mice develop depressive- and anxiety-like behaviors regardless of sex

**Fig. 1:**
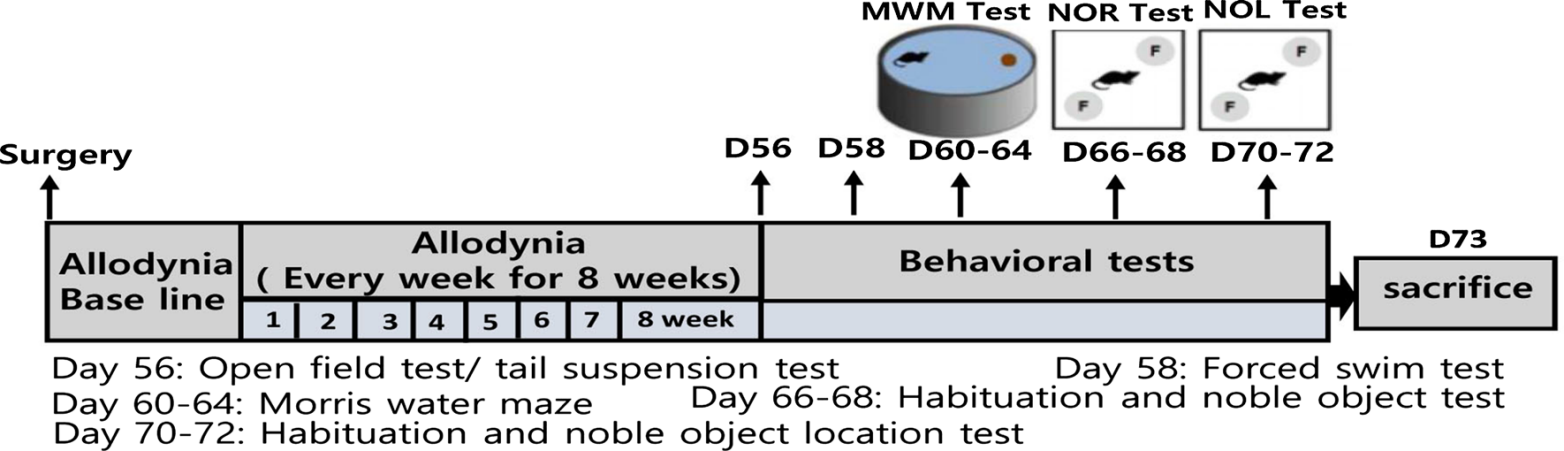
Schematic representation of experimental design for behavioral tests. *MWM* Morris water maze test, *NOR* novel object test, *NOL* novel object location test

**Fig. 2:**
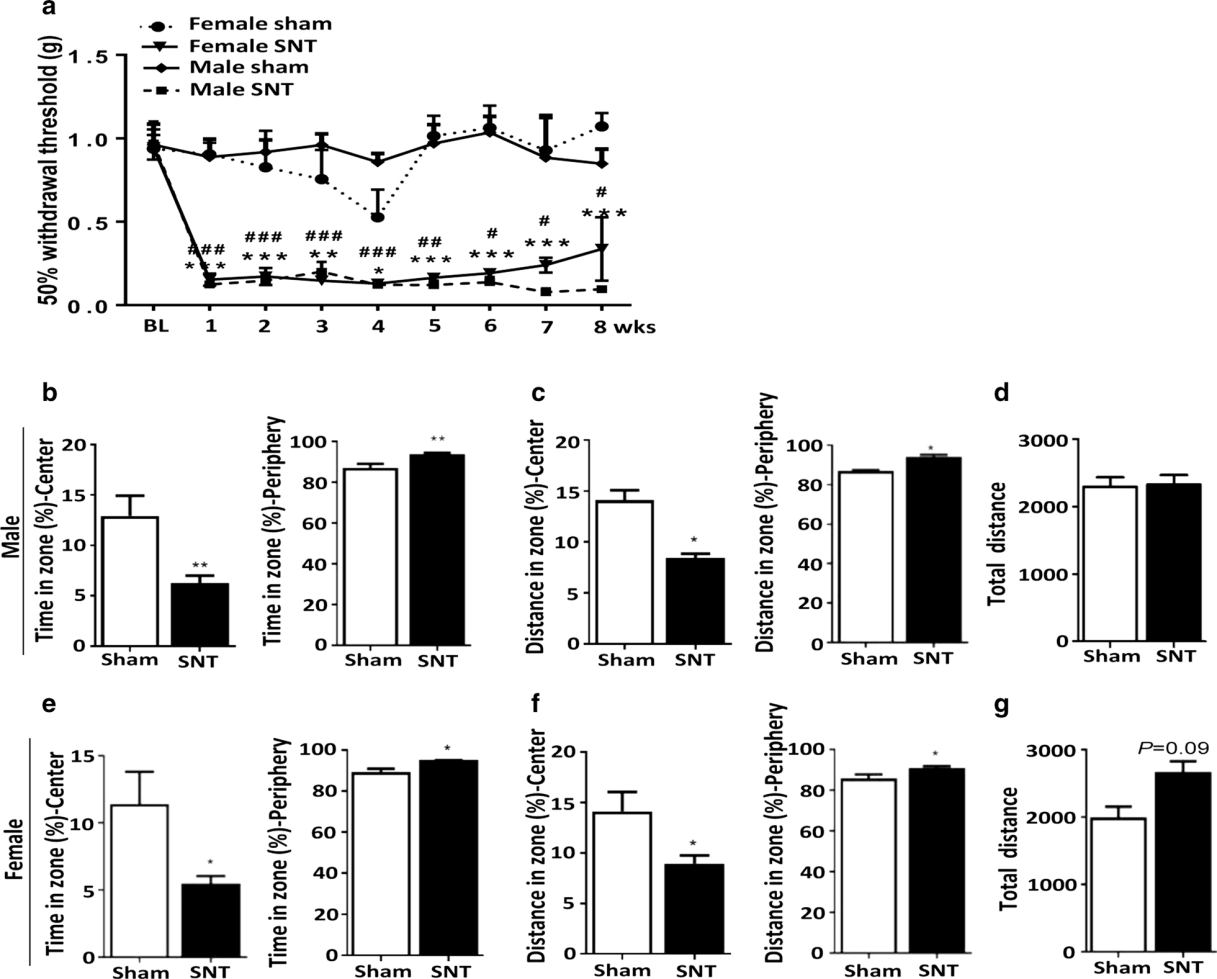
SNT induced anxiety-like behavior in both male and female mice. **a** SNT caused a significant reduction in the hind paw mechanical withdrawal thresholds of both male (n = 8 and 9 for the SNT and sham groups, respectively) and female (n = 7 and 8 for the SNT and sham groups, respectively) mice. **b**, **e** Average percentage of time spent in the center or periphery during an open field test of male and female sham control and SNT-injured mice. Both male and female SNT-injured mice spent less time in the field center and more time on the periphery than male and female sham control mice. **c**, **f** Average percentage of distance moved in the field center or periphery during the open field test. Both male and female SNT-injured mice traveled a shorter distance in the field center and a longer distance on the periphery than the sham control group. **d**, **g** Total distance moved during the open field test. Data are presented as the mean ± SEM, male: n = 9 for sham, n = 12 for SNT; female: n = 7 for sham, n = 8 for SNT. *P < 0.05, **P < 0.01, ***P < 0.001 compared with the sham group

The experimental design of this study is presented in Fig. 1. To investigate putative sexual dimorphism in chronic pain-associated affective and cognitive disorders, we first assessed whether SNT induces comparable levels of chronic pain phenotypes in male and female mice. Mechanical allodynia was assessed by measuring mechanical withdrawal thresholds in von Frey testing of the ipsilateral hind paws in sham control and SNT-injured mice from 1 week until 8 weeks post-injury (Fig. 2a). At 1 week, the 50% withdrawal threshold decreased to 0.15 g and 0.16 g in male and female mice, respectively, which was maintained for up to 8 weeks. There was no significant difference in the withdrawal thresholds between male and female mice during the entire test period (Fig. 2a).

To test whether SNT-induced chronic pain is accompanied by affective disorders in mice, anxiety-like behaviors were first assessed in the open field test. Time spent in the central area in the open field test decreased by 46% and 42% in male and female SNT-injured mice, respectively, compared with sham control mice (Fig. 2b and e, *F* (9, 11) = 6.047, *P* < 0.01, *F* (7, 6) = 21.63, *P* < 0.01). Accordingly, time spent on the periphery increased in both male and female mice (Fig. 2b and e, *F* (9, 11) = 6.047, *P* < 0.01, *F* (7, 6) = 21.63, *P* < 0.01). These are behaviors generally deemed to indicate anxiety. Likewise, the distance moved in the center by the SNT-injured mice decreased significantly (Fig. 2c and f, *F* (8, 11) = 4.698, *P* < 0.05, *F* (7, 6) = 6.773, *P* < 0.05) and that on the periphery increased (Fig. 2c and f, *F* (8, 11) = 4.698, *P* < 0.05, *F* (7, 6) = 6.773, *P* < 0.05) regardless of sex. However, the total distance traveled did not differ between the SNT-injured and sham control mice (Fig. 2d, g), indicating that the behavioral phenotype is not merely a result of motor dysfunction after SNT. Taken together, these results indicate that anxiety-like behavior developed in the SNT-injured mice independent of sex.

**Fig. 3:**
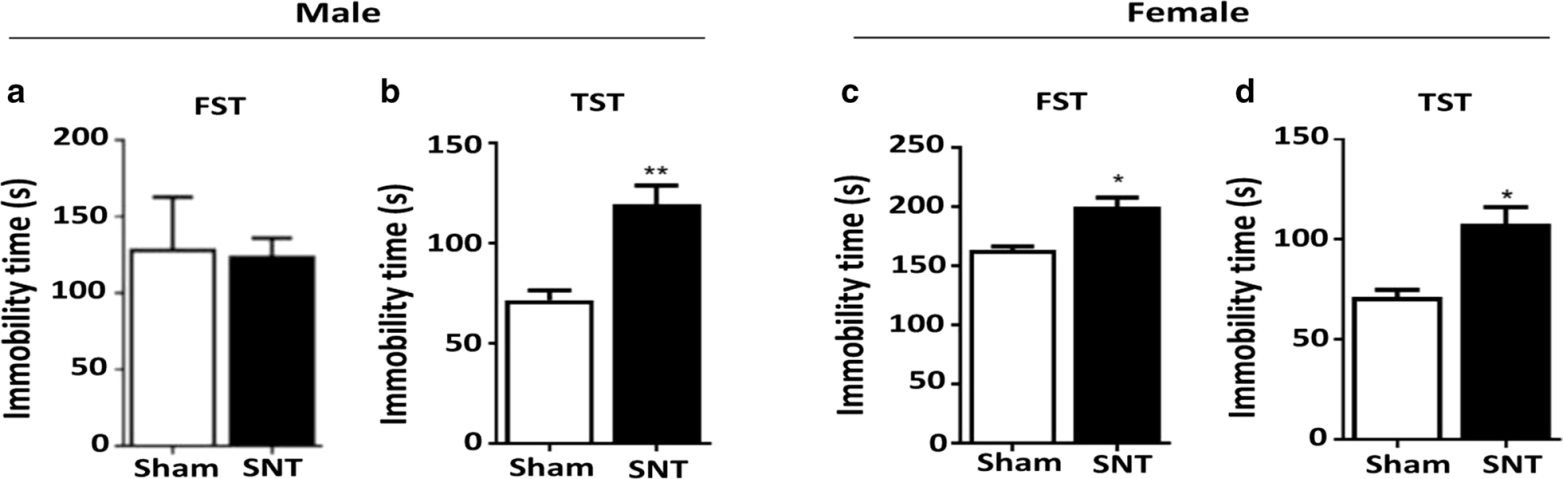
SNT induced depressive-like behavior in both male and female mice. **a**–**d** The forced swim (FST) and tail suspension tests (TST) were used to evaluate depressive-like behavior in male and female mice. The representative bar graph shows the immobility time in the FST and TST 8 weeks after SNT. Data are presented as the mean ± SEM, male: n = 9 for sham, n = 12 for SNT; female: n = 7 for sham, n = 8 for SNT. *P < 0.05, **P < 0.01, ***P < 0.001 compared with the sham group

Depressive-like behaviors were then assessed with the forced swim and tail suspension tests, two well-known behavioral tests used to assess mouse depression [26]. Immobility time in the tail suspension test, an indication of a depressive phenotype, increased in both male and female mice (Fig. 3b, d) 8 weeks after SNT. The increase rate did not differ statistically between these two groups: it increased 45% in male mice and 38% in female mice compared with the sham control mice (Fig. 3, *F* (11, 11) = 3.585, *P* < 0.05, *F* (7, 7) = 5.317, *P* < 0.05). A slight but significant increase in immobility in the forced swim test was observed in SNT-injured female mice but not in male mice (Fig. 3a, c, *F* (7, 7) = 6.697, *P* < 0.05). These data suggest that depressive-like symptoms developed in both male and female mice exposed to chronic neuropathic pain via SNT for 2 months. However, the level of depressive-like behavior might differ between male and female mice depending on the tests performed.

### Sexual dimorphism in chronic neuropathic pain-associated cognitive deficits

**Fig. 4:**
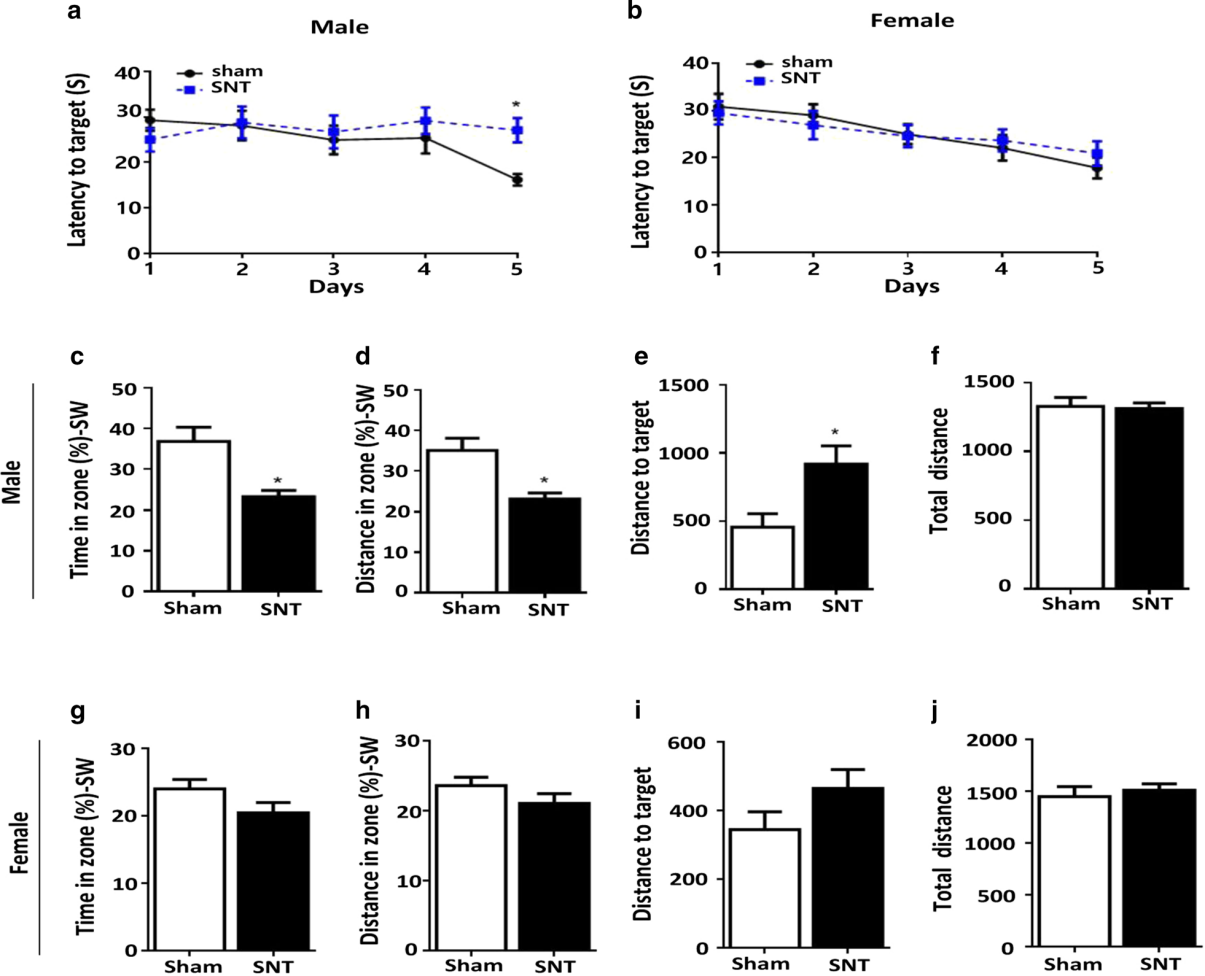
SNT impaired spatial learning memory in male mice but not in female mice. SNT-injured male mice (**a**), but not female mice (**b**), required more time to escape onto the hidden platform compared with the sham group. Performances of sham and SNT-injured male (**c**–**f**) and female (**g**–**j**) mice in a Morris water maze probe trial. Time spent and swimming distance in the target quadrant were recorded. The total distance did not differ between the sham and SNT groups on the probe test. Data are presented as the mean ± SEM., male: n = 12 for sham, n = 12 for SNT; female: n = 12 for sham, n = 12 for SNT. *P < 0.05, **P < 0.01, ***P < 0.001 compared with the sham group

To test whether chronic pain affects cognitive function, mice were subjected to the Morris water maze test to evaluate their spatial learning and memory. In the hidden platform test (Fig. 4a), a significant decrease in escape latency was observed between the sham and SNT groups of males at 5 days. In contrast, no significant difference was observed in escape latency between the sham and SNT groups of females (Fig. 4b). Memory retention was assessed 24 h after the final trial in the absence of the escape platform. In the probe trials, male SNT-injured mice had significantly reduced travel time and distance in the target quadrant area compared with the sham control group (Fig. 4c, d, *F* (8, 11) = 4.929, *P* < 0.05), whereas no significant difference was found between the female sham and SNT groups (Fig. 4g, h). Male SNT-injured mice traveled a greater distance to reach the target quadrant compared with the sham group (Fig. 4e, *F* (8, 11) = 4.44, *P* < 0.05), whereas no significant difference was found between the female sham and SNT mice (Fig. 4i, g).

**Fig. 5:**
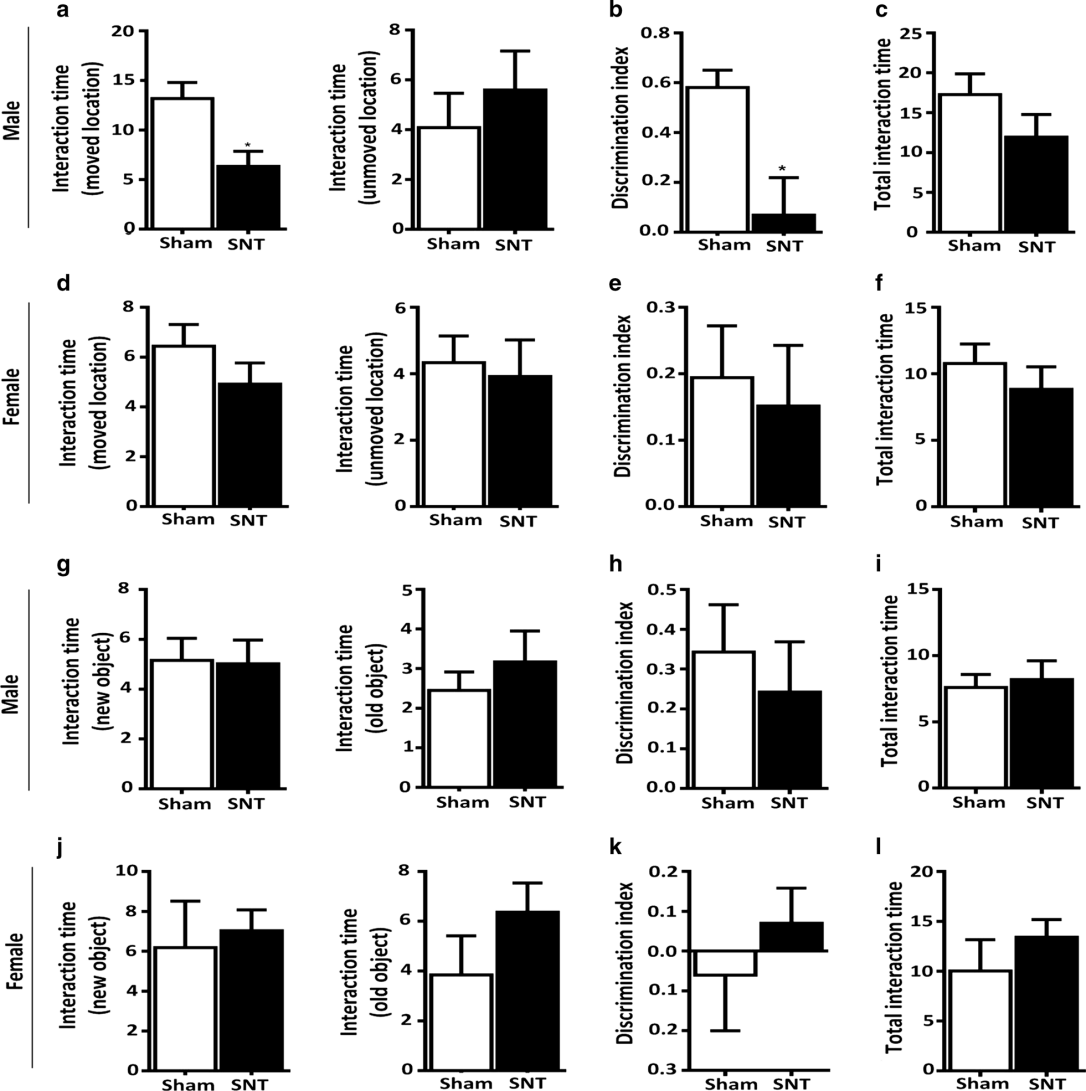
Cognitive deficits measured by the novel object location test were observed in male mice. Interaction time (s) spent exploring the object in the familiar location and the displaced object by male (**a**) and female (**d**) mice. **b**, **e** The discrimination index was defined as (displaced location − familiar location)/(displaced location + familiar location). **c**, **f** Total interaction time spent exploring an object in a familiar location and a displaced object. **g**, **j** Time spent exploring familiar and new objects in the test phase. **h**, **k** The discrimination index was defined as (new object − familiar object)/(new object + familiar object). **i**, **l** Total interaction time spent exploring the familiar object and new object (s). Data are presented as the mean ± SEM, male: n = 12 for sham, n = 12 for SNT; female: n = 12 for sham, n = 12 for SNT. *P < 0.05, **P < 0.01, ***P < 0.001 compared with the sham group

To further confirm a sex-dependent cognitive deficit after SNT, the SNT-injured mice were subjected to an object location recognition test and a novel object recognition test. Again, the male SNT-injured mice showed a higher preference for the object in a familiar place than in a novel place compared with the male sham control mice (Fig. 5a, b). Male SNT-injured mice spent less time interacting with the object in a novel location than the male sham mice (Fig. 5a, *P* < 0.05). Thus, the discrimination index on the interaction time of the male SNT-injured mice in a novel place was significantly lower than that of the sham group (Fig. 5b, *F* (11, 11) = 4.732, *P* < 0.05). In contrast, the discrimination index of the female SNT and sham groups did not differ (Fig. 5d, e). Our analysis of the total exploration duration (novel + familiar location) showed no significant differences between groups in either sex (Fig. 5c–f). For the novel object recognition test, the interaction time did not differ between the sham and SNT groups for male or female mice (Fig. 5g–l). Thus, the discrimination ratio did not differ significantly between the sham and SNT groups of either sex in the novel object recognition test (Fig. 5h, k).

## Discussion

Our aim in this study was to assess whether the affective and cognitive deficits observed in patients with chronic pain are recapitulated in the SNT-induced mouse model of neuropathic pain, and, if so, whether sexual dimorphism exists in those chronic pain-associated affective and cognitive behaviors. Chronic neuropathic pain caused by SNT does lead to depressive- and anxiety-like behavior in mice regardless of their sex, but it induces cognitive deficits only in male mice.

In humans, chronic neuropathic pain is often accompanied by affective disorders such as anxiety and depression that severely impair quality of life and complicate therapeutic approaches. However, the comorbidity of affective disorders and chronic pain has been less pronounced in studies using animal models. Whereas depressive- and anxiety-like behaviors have been observed in some animal models of peripheral nerve injury, as evidenced by increased total immobility time in forced swim or tail suspension tests, other studies have reported no affective component in the dysfunctions caused by chronic pain [27–29]. We noted that those studies tested affective behavior at relatively early time points (within 2 weeks after pain induction), and we reasoned that neuropathic pain-related mood disorders might require a longer time to manifest [30–33]. Therefore, we assessed chronic pain-associated affective and cognitive behavioral abnormalities 8 weeks post-injury and confirmed that depressive- and anxiety-like behaviors indeed develop at that delayed time point (8 weeks post-injury).

The tail suspension and forced swim tests, behavioral assessments commonly used to measure depression in animals, were used to measure chronic pain-associated depression [34]. These tests have been used to evaluate the in vivo efficacy of putative antidepressants for the treatment of pain-related depression [35–38]. In the present study, we found depressive-like behavior in both male and female SNT-injured mice in the tail suspension test (Fig. 3b, d), whereas depressive-like behavior was observed only in female mice in the forced swim test (Fig. 3c). So far, we do not have a clear explanation for the difference in sexual dimorphism of depressive behavior as measured by those two behavioral tests. Perhaps the tail suspension test is less stressful and has higher pharmacologic sensitivity than the forced swim test [39]. The dopaminergic system is reportedly implicated in the performance of mice in the forced swim test, whereas both the serotonergic and dopaminergic systems are involved in the tail suspension test [40]. However, other pharmacologic studies have suggested the involvement of both the serotonergic and noradrenergic systems in the forced swim test as well [41, 42]. It is conceivable that different neuropeptidergic systems are involved in the depressive-like behavior observed in the forced swim and tail suspension tests, and those differences might underlie the different outcomes we found.

Previous clinical studies have suggested that pain might be associated with cognitive impairment in humans and rodents [43]. The results from animal pain models have shown impaired learning and memory reflected through various tasks, such as the Morris water maze [44, 45], novel object recognition [46], and the Y-maze [47]. Consistent with previous data, we observed significant deficits in spatial memory acquisition and retention in the Morris water maze (Fig. 4a, c–f) and in attention toward a novel location (Fig. 5a–c) in male mice after SNT. However, female mice with SNT did not show any changes in those tasks (Figs. 4b, g–j, 5d–f). Recently, sexual dimorphism in cognitive deficits after neuropathic pain was observed in a neuropathic pain mouse model [48]. In that study, male mice were more susceptible than female mice to pain-related cognitive deficits associated with morphologic changes in the parvalbumin interneuron and pyramidal neuron in the infralimbic cortex in males. Similarly, males were more prone to cognitive deficits caused by acute and chronic stress [49–51]. Male-specific alterations in synapses and myelinated axons and decreased µ-opioid receptor signaling have been proposed as possible mechanisms of such sexual dimorphism [50, 52–54]. However, no previous report has considered sexual dimorphism in the cognitive impairment of chronic pain patients, though a higher incidence of cognitive decline has been reported in male patients with Lewy body dementia, Parkinson disease dementia, and vascular dementia [55–58]. Several studies implicate sex hormone levels in cognitive impairment [59–61]. For instance, estrogen has been shown to enhance hippocampus-dependent memory formation [62]. Estrogen replacement therapy is widely used to reduce cognitive deficits in menopausal women [63]. Koyama et al. [64] showed a positive correlation between higher plasma estrogen and better cognitive performance. These results suggest that sex hormones might play a role in the sexual dimorphism of chronic pain-associated cognitive deficits observed in our study. Considering the prior studies, it will be worthwhile to test whether any of the mechanisms they discuss are involved in the sexual dimorphism observed in the Morris water maze and novel object location tests in our study.

Of interest, we found no significant difference between the sham and SNT groups in the novel object recognition test (Fig. 5g–l). The Morris water maze and novel object location tests assess hippocampal-dependent spatial learning and memory [24, 65]. In contrast, the object recognition test relies on a variety of brain regions other than the hippocampus. Therefore, it is possible that chronic pain-induced cognitive deficits in males are associated with a hippocampal abnormality. Indeed, putative functional disturbances in fronto-hippocampal connectivity have been suggested as a relevant cause of pain-involved working memory deficits in patients with pain. A smaller hippocampus was also observed in elderly patients with chronic pain. Hippocampal abnormalities associated with short-term memory deficits [66], recognition memory deficits [46], and deficits in long-term potentiation [67] have been reported in animal models of neuropathic pain. Considering the putative involvement of the hippocampus in chronic pain-associated cognitive dysfunctions, it would be interesting to test whether hippocampal neurons are differentially affected by chronic pain depending on sex. That idea warrants future investigation.

## Conclusion

Our study found that SNT-induced neuropathic pain led to depressive- and anxiety-like behavior and cognitive deficits at a delayed time point in mice. Furthermore, we found sexual dimorphism in chronic pain-associated cognitive deficits. These results provide an animal model of sexually dimorphic cognitive deficits, and future studies could elucidate the pathogenic mechanisms of cognitive deficits observed in neuropathic patients.

## Data Availability

Not applicable.

## Abbreviation

SNT: L5 spinal nerve transection
